# A prospective, multicenter, real-world observational study evaluating the impact of tibial runoff on clinical outcomes after endovascular therapy for femoropopliteal lesions: Research protocol

**DOI:** 10.3389/fcvm.2022.1035659

**Published:** 2022-11-16

**Authors:** Yang Liu, Qiqi Wang, Ziheng Wu, Zibo Fen, Lianrui Guo, Qiang Li, Xin Fang, Hongfei Sang, Yudi Dai, Chunshui He, Meng Ye

**Affiliations:** ^1^Department of Vascular Surgery, Hospital of Chengdu University of Traditional Chinese Medicine, Chengdu, China; ^2^Department of Vascular Surgery, The First Affiliated Hospital of Medicine College of Zhejiang University, Hangzhou, China; ^3^Department of Vascular Surgery, Liyuan Hospital Affiliated Tongji Medical College of Huazhong University of Science and Technology, Wuhan, China; ^4^Department of Vascular Surgery, Xuanwu Hospital Capital Medical University, Beijing, China; ^5^Department of Vascular Surgery, Qingdao Haici Hospital Affiliated to Qingdao University, Qingdao, China; ^6^Department of Vascular Surgery, School of Medicine, Affiliated Hangzhou First People's Hospital, Zhejiang University, Hangzhou, China; ^7^Department of Vascular Surgery, The Second Affiliated Hospital of Soochow University, Suzhou, China; ^8^Biomedical Informatics and Statistics Center, Fudan University School of Public Health, Shanghai, China; ^9^Department of Vascular Surgery, Renji Hospital, School of Medicine, Shanghai Jiaotong University, Shanghai, China

**Keywords:** arteriosclerosis obliterans, endovascular intervention, femoropopliteal lesion, tibial runoff, protocol

## Abstract

**Introduction:**

Current evidence indicates endovascular intervention is a safe and effective treatment for peripheral artery disease of the lower extremity. However, the clinical outcome of endovascular intervention for femoropopliteal lesions has been shown to be affected by the status of tibial runoff. It remains unclear whether endovascular intervention for tibial runoff is associated with additional benefits.

**Methods and analysis:**

This prospective, multicenter, real-world observational study is carried out from January 2021 to December 2022 in 8 designated centers across China with an estimated sample size of 1200 patients with severe femoropopliteal disease. The pre-procedural status of tibial runoff is evaluated with the modified SVS score and categorized as good (SVS <5), compromised (SVS 5–10) or poor (SVS >10). Whether the patient will be treated with endovascular intervention for tibial runoff is determined by the treating vascular surgeons. Patients are dichotomized into the intervention group and the non-intervention group, with each group further divided into the good, compromised and poor tibial run-off subgroup, yielding 6 subgroups in total. Patients within various subgroups are compared with regard to the primary patency rate of the femoropopliteal artery, changes in quality of life, changes of Rutherford category, improvement of the Wound, Ischemia, and Foot Infection Classification, and incidence of major adverse events over 24-months follow-up. The results of this study may provide important information to help vascular sspecialists to decide whether the tibial runoff should be endovascularly intervened and which patient population benefits most from tibial runoff intervention.

**Clinical trial registration:**

https://clinicaltrials.gov/ct2/show/NCT04675632?id=NCT04675632&draw=2&rank=1, NCT04675632.

## Introduction

Peripheral artery disease (PAD) of the lower extremity is a common occlusive vascular disease that most frequently affects the middle-aged and the elderly. As a presentation of atherosclerosis, PAD predominantly involves the abdominal aorta, as well as the small and medium-sized arteries of the lower extremities that contributes to claudication and chronic limb-threatening ischemia. It is reported that the overall prevalence of PAD in the general population is about 3–10%, which is increased to 15–20% in people aged over 70 (1). Current management strategies for PAD include pharmacotherapy, open surgery, endovascular therapy and exercise therapy (2). Of note, endovascular therapy for lower extremity PAD has been shown to be a safe and effective procedure associated with a lower rate of patient morbidity and mortality (3). Therefore, endovascular therapy has been increasingly used as the first-line treatment modality for PAD of the lower extremity where revascularization is indicated.

Although the majority of studies have shown that poor infrapopliteal run-off had a negative impact on the clinical outcome following femoropopliteal artery reconstruction, the implications of distal runoff in predicting patency after superficial femoral artery (SFA) angioplasty remains unclear. Currently, there are still controversies with regard to the relationship between distal run-off and primary patency after femoral-popliteal interventions. Recently, Noh et al. (4) showed the primary patency rate after stenting in SFA was significantly lower in the compromised run-off group at 36 months, as compared with the good run-off group (49.8 vs. 64.6%, *P* = 0.011). Similar findings were reported by Hiramori et al. (5). On the contrary, the studies by Lee's group (6) and Wilson's group (7) reported that tibial runoff did not influence the primary patency after bare-metal stenting of the femoral and popliteal arteries.

In addition, it should be noted that most of the previous studies excluded patients who underwent concomitant run-off angioplasty to avoid bias on analysis (5, 8). Is it possible to improve the primary patency rate after treatment for femoropopliteal lesions by aggressively performing run-off reconstruction? Watanabe and colleagues retrospectively analyzed data from a total of 238 limbs in 199 patients with *de novo* SFA chronic total occlusion lesions treated with bare-metal nitinol stents (9). Patients in the subgroup with additive tibial percutaneous transluminal angioplasty had significantly higher major adverse limb events-free survival rate at 2 years compared with those without tibial percutaneous transluminal angioplasty (65.5 vs. 26.2%; *P* = 0.001). Although no further imaging evaluation between the two groups were performed, the evidence highlighted a potential improvement of SFA stenting by active treatment of concomitant tibial lesions, even in intermittent claudicants. However, in the guideline from the European Society of Cardiology Guidelines on Peripheral Artery Disease in collaboration with the European Society for Vascular Surgery, primary tibial artery angioplasty is not recommended (10). The symptoms could become even worse after tibial artery angioplasty in case of technical failure. Therefore, the decision of concomitant tibial angioplasty should be carefully evaluated until long-term data is available.

For these reasons, we aim to conduct a prospective real-world observational study with more subgroup analysis that focuses not only on the impact of tibial run-off on clinical outcomes after femoropopliteal intervention, but also the impact on the patency after the treatment for femoropopliteal lesions.

## Objective

This study aims to bridge the gap in the role played by tibial runoff in endovascular treatments for femoropopliteal lesions. Thus, the objective is to assess whether primary endovascular intervention for tibial runoff in conjunction with femoropopliteal disease could provide additional significant beneficial outcomes as compared with endovascular intervention for femoropopliteal disease alone.

## Methods and analysis

### Study design

This is a prospective, multicenter, real-world, observational clinical study carried out from January 2021 to December 2022. The study population includes 1,200 patients with chronic severe femoropopliteal disease that are recruited from eight designated centers in China.

The study consecutively enrolls, over an approximate 2-year period, patients with severe femoropopliteal disease who are eligible for endovascular therapy. Whether the patient will be intervened for tibial runoff is at the discretion of the treating vascular surgeons based on the status of tibial runoff. The pre-procedural and post-procedural status of tibial runoff is evaluated with the modified Society of Vascular Surgery (SVS) runoff scoring system with a maximal score of 19. In general, a score of <5, 5–10 and >10 is categorized as good, compromised and poor tibial runoff (11).

The choice of the modified SVS score over other scoring systems, such as the Global Limb Anatomic Staging System (GLASS), is based on the notion that the GLASS is generally applied to evaluate the single target arterial path, and is not suitable for assessing the definitive status of 3 tibial run-offs (12). In fact, the modified SVS run-off score has been widely adopted for quantitative analysis in prior reports (13–17).

Patients are divided into the following six groups based on the pre-procedural status of tibial runoff and whether the tibial runoff is intervened:

Group 1A: good tibial runoff with runoff intervention;

Group 1B: good tibial runoff without runoff intervention;

Group 2A: compromised tibial runoff with runoff intervention;

Group 2B: compromised tibial runoff without runoff intervention;

Group 3A: poor tibial runoff with runoff intervention;

Group 3B: poor tibial runoff without runoff intervention.

### Sample size estimation

Based on the results of previous studies (5, 9, 16, 18, 19) and physician experience, we assume patency rates of 80 and 65%, 75 and 60%, and 50 and 30% in the intervention and non-intervention groups, respectively, in the good, compromised and poor tibial runoff group. Assuming 1-sided α = 0.025 and β = 80%, at least 432, 365 and 200 subjects are required for the good, compromised and poor tibial runoff group, respectively, to account for a potential 20% attrition rate. Consequently, a minimum sample size of 997 in total are required. For this study, a total of 1,200 patients with chronic severe femoropopliteal disease are recruited by taking into consideration the enrollment ability of the participating centers.

### Eligibility criteria

#### Inclusion criteria

To be eligible to participate in this study, a subject must meet the first three of the following criteria:

1) Presence of severe stenosis (≥70%) or occlusive disease involving femoropopliteal artery.

2) Subjects must understand the purpose of the study and are willing to participate in follow-up visits.

3) Subjects must give informed consent.

4) Subjects can also be enrolled in the presence of the following special scenarios:

a. Patients with restenosis following a previous endovascular intervention for femoropopliteal disease.

b. Femoropopliteal diseases involving both lower extremities.

c. Patients with a previously technically failed endovascular treatment but a later successful endovascular intervention.

d. Presence of concomitant ipsilateral aortoiliac lesions with a residual stenosis <30% after endovascular intervention.

#### Exclusion criteria

A subject who meets any of the following criteria will be excluded from participation in this study:

1) Patients with acute arterial thrombotic events.

2) Serum creatinine higher than 2 mg/dL.

3) Rutherford category of 5 with an infection score of 2 or 3 as determined by the Wound, Ischemia, and Foot Infection classification.

4) Patients with a previous history of femoral and popliteal bypass.

5) Known allergic history to contrast agents, paclitaxel, anticoagulant or antiplatelet medications.

6) Patients with coagulation abnormalities.

7) Pregnant or lactating women.

8) Patients with unstable angina, myocardial infarction, transient ischemic attack or stroke within the past 3 months.

9) Patients with concomitant severe diseases, such as liver failure.

10) Patients with a life expectancy <24 months.

11) Patients who participate in other clinical trials during the same period.

12) Patients with poor compliance, defined as continued smoking after the procedure.

3.4 Withdrawal of individual subjects/Termination criteria

Subjects can withdraw from the study at any time for any reason without compromising future treatment. The investigator can also decide to withdraw a subject from the study for the following medical reasons:

1) Safety and ethical considerations, such as serious adverse events.

2) The patient is lost to follow-up.

3) The patient voluntarily withdraws the informed consent.

4) Serious violation of the protocol by the subject or the investigator.

5) The investigator considers it necessary to withdraw a subject from the study for other reasons.

6) Early termination index is reached.

For patients who withdraw from the trial, attempts will be made to retrieve data on both the primary and secondary outcomes.

### Interventions

All patients are treated with endovascular therapy with either the contralateral femoral artery retrograde approach, the ipsilateral femoral artery antegrade approach or the brachial artery approach. If antegrade endovascular treatment fails in the process of antegrade recanalization of target lesions, retrograde puncture at the distal end of the lesion can be considered for bidirectional recanalization. After the guide wire passes through the lesion, the operator can independently determine the treatment scheme according to the characteristics of the lesion with either plain balloon angioplasty alone, plain balloon angioplasty + stent implantation, drug-coated balloon angioplasty, drug-coated balloon angioplasty + stent implantation, or atherectomy + drug-coated balloon angioplasty.

### Outcome measures

The main outcomes assessed is the primary patency rate of the femoropopliteal artery, which is defined as the absence of clinically-driven target lesion revascularization and/or recurrent target lesion stenosis ≥50% by imaging (e.g., invasive angiography or, most commonly, duplex ultrasonography) (20).

The secondary outcomes include changes in quality of life, changes of Rutherford category, improvement of the Wound, Ischemia, and Foot Infection Classification, and incidence of major adverse events. The Vascular Quality of Life Questionnaire (21), which evaluates health-related quality of life from domains of symptoms, pain, activities, social and emotional, is applied to quantify patient's quality of life. Major adverse events include disease-related mortality, major amputation, and acute vascular events that included myocardial infarction, ischemic stroke and acute limb ischemia (22).

### Timeline of visits

The study follow-up period is expected to be 24 months. According to published literature and relevant clinical practice experience, the patient is expected to complete the following follow-up visits:

Pre-enrollment visit: Patients who provided signed informed consent are assessed with regard to personal data and complete clinical history. Subsequently, preoperative laboratory tests that include complete blood count, liver and kidney function tests, blood lipid profiles, coagulation assessment and electrocardiogram are conducted.

Baseline visit (Day of operation/Day 0): Patient vital signs, clinical symptoms and surgical data are collected. After completion of the surgical arteriography, patient eligibility is determined by the investigator. For those eligible, the pre- and post-procedural runoff score, technical success and adverse events are recorded.

3. Postoperative visit (Day 1– Day 21): Data regarding patient quality of life score, ankle-brachial index, Doppler ultrasonography examination results and medications are obtained.

4. Follow-up 1 to follow-up 5: In these follow-ups on 1 month ± 7 days, 6 months ± 30 days, 12 months ± 30 days, 18 months ± 30 days and 24 months ± 30 days, respectively, patient quality of life score, ankle-brachial index, Doppler ultrasonography examination results and medications are obtained.

### Data collection

A standard case report form (CRF) was designed at the beginning of this study. This form is used to obtain demographic information, disease history, surgical details, data elements, imaging features, and outcome events for study participants. Data is primarily recorded on paper CRFs, and two researchers will input the CRFs to an electronic database simultaneously to avoid typing errors.

Investigators are responsible for ensuring that the CRFs and original medical records are filled in completely and accurately. Each CRF and original medical record should only record the data of one patient. Any incorrect data or words cannot be altered but should be marked out with a single line and filled in again with the correct data or words on the side, along with the signature of the investigator and the current date. The original medical record is kept at each center. The original CRF is kept by the sponsor and a copy of the CRF is kept at each center.

In order to minimize the dropout rate, this study is carried out with the collaborative efforts of vascular surgeons from eight university affiliated hospitals. Furthermore, an electronic system called “Vessel Health” was created, and the patient information is recorded immediately after surgery by two professionals. At last, a professional contract research organization is hired to ensure quality control, monitoring and coordination.

### Adverse events recording, evaluation, analysis, and reporting

The investigator should record all adverse events that occur during the clinical trial. In addition, the investigator should evaluate and analyze the causes of these events together with the sponsor in the form of a written report. The investigator should discuss opinions on the continuation, suspension, or termination of the trial. These reports are submitted to the Ethics Committee for review by the management department of clinical trials.

In the case of serious adverse events that occur during the clinical trial, the investigator should immediately take appropriate treatment measures for the subjects and report this in a written format to the clinical trial management department. In addition, the investigator should notify the sponsor with a written form. The medical device clinical trial management department reports this in writing to the corresponding ethics committee, which is under the supervision and management of the site where the clinical trial institution is located, and the health and family planning department within 24 h of the event. For an event that has resulted in death, the clinical trial institution and the investigator should provide all necessary information to both the ethics committee and the sponsor.

### Statistical analysis

All statistical analyses performed are guided by the intention-to-treat principle. Enumeration data is expressed as number (percentages) and compared using chi-squared test, Fisher's exact test, or Cochran-Mantel-Haenszel chi-squared test, as appropriate. The McNemar test is used for intra-group comparisons. Measurement data is compared between groups using non-parametric Wilcoxon tests or one-way analysis of variance with the *post-hoc* Tukey test.

Considering the possible confounding and bias in real-world studies, generalized linear model and multivariate analysis strategy are adopted based on propensity score. A two-sided *P*-value < 0.05 denotes statistical significance.

## Discussion

According to prior publications (4, 5), the status of tibial runoff plays a critical role for the clinical outcomes following endovascular treatment for femoropopliteal lesions. Nonetheless, whether endovascular treatments for tibial runoff in conjunction with femoropopliteal disease could provide additional clinical benefits as compared with those without tibial runoff intervention remain uninvestigated in prospective, real-world studies. The present study represents an attempt to bridge this gap by exploring the clinical benefits of tibial runoff endovascular intervention in terms of freedom from CD-TLR, the primary patency rate of the femoropopliteal artery, changes in quality of life, changes of Rutherford category, improvement of the Wound, Ischemia, and Foot Infection Classification, and incidence of major adverse events. The results of this study may provide important information to help vascular specialists to decide whether the tibial runoff should be intervened and which patient population benefits most from tibial runoff intervention.

## Ethics statement

The study protocol has been approved by the Medical Ethics Committee of the Affiliated hospital of Chengdu University of Traditional Chinese Medicine (No. 2020KL-078) and is conducted in accordance with the provisions of the Declaration of Helsinki. The study protocol has been registered at Clinicaltrials.gov (Identifier NCT04675632). Ultimately, we aim to publish the results of this study in a peer-reviewed journal.

## Author contributions

CH and QW contributed to the conception and design of the study, drafted the protocol, and supervised the revision. LG, YL, ZW, ZF, QL, XF, and HS provided intellectual input to improve the study design and revise the protocol. MY and ZW supervised the conception and design of this study. All the authors read and approved the final manuscript.

## Conflict of interest

The authors declare that the research was conducted in the absence of any commercial or financial relationships that could be construed as a potential conflict of interest. Reviewer XC declared a shared affiliation with two of the authors MY and YD to the handling editor at time of review.

## Publisher's note

All claims expressed in this article are solely those of the authors and do not necessarily represent those of their affiliated organizations, or those of the publisher, the editors and the reviewers. Any product that may be evaluated in this article, or claim that may be made by its manufacturer, is not guaranteed or endorsed by the publisher.
